# A Multiarmed Bandit Approach to Adaptive Water Quality Management

**DOI:** 10.1002/ieam.4302

**Published:** 2020-08-14

**Authors:** David M Martin, Fred A Johnson

**Affiliations:** ^1^ Maryland/DC Chapter, The Nature Conservancy Bethesda Maryland USA; ^2^ Department of Bioscience Aarhus University Ronde Denmark

**Keywords:** Adaptive management, Bayesian, Decision making, Nitrogen monitoring

## Abstract

Nonpoint source water quality management is challenged with allocating uncertain management actions and monitoring their performance in the absence of state‐dependent decision making. This adaptive management context can be expressed as a multiarmed bandit problem. Multiarmed bandit strategies attempt to balance the exploitation of actions that appear to maximize performance with the exploration of uncertain, but potentially better, actions. We performed a test of multiarmed bandit strategies to inform adaptive water quality management in Massachusetts, USA. Conservation and restoration practitioners were tasked with allocating household wastewater treatments to minimize N inputs to impaired waters. We obtained time series of N monitoring data from 3 wastewater treatment types and organized them chronologically and randomly. The chronological data set represented nonstationary performance based on recent monitoring data, whereas the random data set represented stationary performance. We tested 2 multiarmed bandit strategies in hypothetical experiments to sample from the treatment data through 20 sequential decisions. A deterministic probability‐matching strategy allocated treatments with the highest probability of success regarding their performance at each decision. A randomized probability‐matching strategy randomly allocated treatments according to their probability of success at each decision. The strategies were compared with a nonadaptive strategy that equally allocated treatments at each decision. Results indicated that equal allocation is useful for learning in nonstationary situations but tended to overexplore inferior treatments and thus did not maximize performance when compared with the other strategies. Deterministic probability matching maximized performance in many stationary situations, but the strategy did not adequately explore treatments and converged on inferior treatments in nonstationary situations. Randomized probability matching balanced performance and learning in stationary situations, but the strategy could converge on inferior treatments in nonstationary situations. These findings provide evidence that probability‐matching strategies are useful for adaptive management. *Integr Environ Assess Manag* 2020;16:841–852. © 2020 The Authors. *Integrated Environmental Assessment and Management* published by Wiley Periodicals LLC on behalf of Society of Environmental Toxicology & Chemistry (SETAC)

## KEY POINTS

- Adaptive water quality management can be improved with statistically designed experimentation.
- Multiarmed bandit strategies are specialized statistical experiments to inform the adaptive management of uncertain actions.
- Findings provide evidence that probability‐matching strategies can inform adaptive water quality management.

## INTRODUCTION

Total maximum daily load policies in the United States Clean Water Act require states to identify impaired waters that are not compliant with water quality criteria. States are responsible for meeting water quality criteria; whereas local conservation and restoration organizations are tasked with implementing point and nonpoint source management actions to reduce pollutant inputs to impaired waters. The primary goal of these actions is to restore vital habitats for recreational fishing, wildlife, and infrastructure.

A fundamental problem in water quality management lies in deciding how to allocate point and nonpoint source actions and monitor desired environmental outcomes (Adler et al. 1993). To support this process, adaptive management is a learning practice to allocate actions based on prior scientific observations, learn about the actions, and replicate the allocation process with new information (Holling 1978; Walters 1986). Adaptive management has been extensively used to facilitate water quality management where uncertainty and variability are key aspects of the problem (e.g., National Research Council 2001; Gunderson and Light 2006; Kingsford and Biggs 2012). One of the most challenging aspects of adaptive management is deciding the best allocation strategy to maximize performance, reduce uncertainty, and ultimately improve environmental outcomes (Robbins 1952). The US Department of the Interior approach to adaptive management integrates monitoring data and statistical methods, which allows the allocation of actions to become more easily predicted and results in reduced uncertainty over time (Williams and Brown 2012).

**Figure 1:**
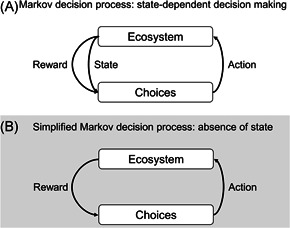
(**A**) Markov decision process and (**B**) simplified Markov decision process.

Adaptive management is often framed as a Markov decision process, where the choice of action depends on the state of the managed ecosystem at each decision epoch (so‐called “state‐dependent” decisions; Figure 1A). Actions generate an immediate reward (i.e., net benefit as measured by performance metrics) and, along with other uncontrolled factors, drive the system to a new state. Actions affect both the current reward and the probability that the ecosystem will transition to a new state (McCarthy and Possingham 2007; Williams 2011).

In some problem formulations, the most appropriate actions are not state‐dependent (Figure 1B). The immediate reward is again linked to the implemented actions, but here the decision maker chooses actions that are most likely to yield the best reward in the absence of its effect on state transitions. This is a general problem for conservation and restoration organizations, who often implement multiple nonpoint source actions in the absence of monitoring long‐term or large‐scale environmental outcomes. State water quality managers are faced with limited monitoring budgets, and there are time lags between nonpoint source actions and environmental responses. State‐sponsored water quality models may not be able to predict the movement of individual pollutants in water from multiple nonpoint sources. Furthermore, they may not contain information that can support site‐ and source‐specific environmental determinations that link an action's performance to the desired ecosystem state (Boyd 2000).

How do we implement nonpoint source management actions and monitor their performance in the short term to improve estuary properties in the future? In other words: what is the strategy to implement the best actions over time? We encounter these types of questions from conservation and restoration practitioners at different scales and levels of partnership. If the best action is the priority under uncertainty, then adaptive management can be framed as a multiarmed bandit problem.

The term multiarmed bandit is a metaphor for a slot machine problem, where casino gamblers must choose which slot machine arm to play to achieve the highest payoff (Scott 2010). To acquire the highest payoff, the gambler must make sequential choices that can either exploit 1 arm, the arm that currently performs “best,” or explore multiple arms to cover areas of uncertainty and possibly discover better‐performing arms. Therefore, the gambler is faced with a tradeoff between exploitative decisions that could maximize short‐term rewards and explorative decisions that could correctly identify the superior arms to maximize payoffs over the long term. The tradeoff exists because exploitative decisions act to limit explorative decisions (Villar et al. 2015). In other words, decisions that tend to exploit what is currently known about an action's performance can limit the exploration of other actions that could prove superior in the end. Many multiarmed bandit strategies have been used in medicine (Villar et al. 2015), education (Rafferty et al. 2019), behavioral science (Eckles and Kaptein 2019), software design in e‐commerce (e.g., Amazon, Google, eBay) (Scott 2010; Cai et al. 2018), and species management (Springborn 2014).

Scott (2010) provides a helpful review of optimal and heuristic algorithms to inform multiarmed bandit problems. Of the algorithms reviewed by Scott (2010), only the Gittens index (Gittins 1979) carries a guarantee of optimality, but then only under restrictive conditions that can make problems computationally unwieldy to solve. It is also possible to derive an optimal solution by posing the problem as a Markov decision process where the system is not a physical one, but one that is characterized by a distinct knowledge of an action's performance. For example, where multiple actions have a binary outcome, the system can be described as a series of beta distributions whose parameters are defined and updated by the number of action “successes” and “failures” (Villar et al. 2015). A related approach might use a Dirichlet distribution, the multinomial counterpart of the beta distribution, to express the frequencies with which actions perform “best.” These approaches are difficult to implement and can quickly become computationally intractable for practitioners with limited experience with these concepts.

Heuristic multiarmed bandit strategies have been used extensively. One popular heuristic strategy based on upper‐confidence bounds can be asymptotically optimal as decision making nears an infinite time horizon (Lai and Robbins 1985), but the tractability and generality of the method can be limited (Scott 2010). In contrast, a large number of heuristic strategies do not require restrictive conditions, can be implemented by nonexpert practitioners, and have been shown to provide acceptable performance. Based on the review by Scott (2010) and empirical and theoretical trials (Scott 2010; Chapelle and Li 2011; Agrawal and Goyal 2012; Russo and Van Roy 2014), we examined 2 probability‐matching heuristics and tested their performance against a nonadaptive equal allocation heuristic (see *Methods* section).

### Case study

This article aims to test multiarmed bandit strategies to inform adaptive water quality management problems. We used hypothetical experimentation to test probability‐matching and equal‐allocation strategies using time series of N effluent data from innovative household wastewater treatment types in southeastern Massachusetts, USA, including Cape Cod, Martha's Vineyard, and the Nantucket islands. Water quality in these areas is degraded to the extent that dozens of estuaries have been listed as N‐impaired (Cape Cod Commission 2015). For the past 20 years, the state‐sponsored Massachusetts Estuaries Program (https://www.mass.gov/guides/the-massachusetts-estuaries-project-and-reports) has been developing estuary models and total maximum daily loads in impaired waters to achieve desirable estuary properties, including N concentrations, eelgrass, and benthic fauna habitats.

Thousands of residential homes are on conventional septic systems for waste treatment and disposal. Innovations to conventional septic systems specialize in removing N from household wastewater (Barnstable County 2020). Town and nongovernmental organizations must choose and implement multiple N‐removing wastewater treatment types to minimize N effluent without full information on their performance or effectiveness. Currently, there is no formal decision‐making process for screening the possible innovations to implement total maximum daily load policies. Likewise, there are uncertainties about which treatment types are “best” in terms of N‐removing performance (Martin and Johnson 2019; Martin et al. 2019) as well as discrepancies in monitoring environmental outcomes. The estuary models are not able to track the movement of N in groundwater from individual treatments to environmental outcomes in the estuaries.

This context presents a problem. Monitoring treatment performance is the focus of near‐term decision making, whereas monitoring estuary properties is the focus of long‐term decision making, but these contexts may not be correlated. Ad hoc strategies could limit their effectiveness in the long term. We inform this problem by testing which multiarmed bandit strategies could be useful to make adaptive decisions under different assumptions.

## METHODS

### Treatment data

The Barnstable County Department of Health and Environment maintains a database of total N (sum of kjeldahl N, NO_2_, and NO_3_) effluent concentrations (mg/L) from several N‐removing wastewater treatment types (see Supplemental Data). We obtained data for 3 of the most popular treatment types for the years 2000 to 2018. We refer to these treatment types as Treatment A, Treatment B, and Treatment C to avoid the appearance of endorsing a treatment type. The choice of treatment type was based on maximizing the amount of data available for experimentation. The treatment types differ by proprietary technologies to promote bacterial nitrification and denitrification. The technologies are integrated as a retrofit to a standardized household wastewater septic system. Detailed information and experiments on the differences between the treatment types is respectively provided online (Commonwealth of Massachusetts 2020) and in the literature (Lancellotti et al. 2017; Amador et al. 2018).

There were approximately 220 household installations of Treatment A from 2003 to 2018 (mean N effluent concentration of installations over time is 12.7 mg/L), 152 of Treatment B from 2002 to 2018 (mean N effluent concentration of installations over time is 23 mg/L), and 597 of Treatment C from 2000 to 2018 (mean N effluent concentration of installations over time is 18.2 mg/L). According to these data, we assumed that Treatment A is the superior treatment in the case study.

The Barnstable County Department of Health and Environment database organizes data based on the date at which N concentrations are provided by treatment system operators and uploaded into the database (see Supplemental Data). It is unclear when exactly a household treatment was installed based on the recorded date. According to the results reported to the database, the mean N effluent concentration of Treatment A installations changes significantly over time. The mean N effluent concentration of the first 33 installations recorded between 2003 and 2007 is approximately 22 mg/L, whereas the mean N effluent concentration of the 187 installations recorded between 2008 and 2018 is approximately 11 mg/L. As a result, the time series of Treatment A appears nonstationary regarding the mean N effluent concentration. This is due to a recent upload of data into the database tracking system. However, we assumed that all treatments are stationary, and the mean N effluent concentration or “performance” of the treatment should not change over time.

### Experiments and sensitivity analysis

**Figure 2:**
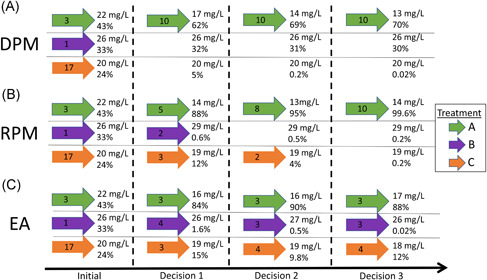
Visualization of multiarmed bandit strategies. One case study experiment using the stationary data set is shown to exemplify how the strategies sample treatments. The horizontal axis refers to the decision, where the initial iteration and first 3 decisions are depicted. Each arrow contains the number of installations sampled per treatment type per decision, followed by its cumulative N effluent concentration (mg/L) and the probability of success (converted to %). The sensitivity iteration shown uses 3, 1, and 17 starting values for Treatments A, B, and C to initiate the experiment. A space with no arrow means that no treatments were sampled in that decision. Exploitation is represented in (**A**), exploration is represented in (**C**), and a balance of exploitation and exploration is presented in (**B**). Reference *Results and Discussion* for further explanation and key findings from this example. *EA—equal allocation; DPM—deterministic probability matching; RPM—randomized probability matching.

The treatment data are based on past household observations. Conservation and restoration practitioners are seeking guidance on how to use the data to make adaptive choices based on which treatments are better for cleaning up Cape Cod estuaries. We designed experiments to sample from the observational data using multiarmed bandit strategies as a test on which strategy performs better concerning 1) minimizing N effluent concentration and 2) the probability of being the superior or “best” treatment (i.e., “probability of success”) over 20 sequential decisions (Figure 2). The experiments were performed to illustrate how to implement total maximum daily load policies over time in an efficient and statistically robust manner. It is important to note that multiple management objectives can be considered in this type of analysis, such as treatment costs. However, we did not include costs in the case study to maintain simplicity with content and the discussion of novel topics. Likewise, evaluating multiple objectives using optimization strategies is beyond the scope of this article (see *Results* section).

We developed 2 different data sets to sample the observational data. A nonstationary data set sampled the observational data based on how they were recorded in the Barnstable County database, such that the mean N effluent concentration of Treatment A appears to change throughout the installations. We randomly selected data in a stationary data set without regard to the date of acquisition, such that each treatment's mean N effluent concentration does not appear to change throughout the installations.

The experiments were designed to sample 10 installations each in a sequence of 20 decisions (200 total installations were sampled or “allocated,” per experiment). Each installation had 1 or more N effluent concentrations over the years. Also, there were gaps in data for many of the allocations. Over these gaps in available data, we assumed that a single installation's performance was equal to its mean N effluent concentration (mg/L) over the life cycle of the installation (from date of installation to 2018). We chose 20 decisions to compare the performance and learning of each strategy and address limitations in the data sets. We performed 6 sensitivity analyses per strategy per experiment. Each sensitivity analysis had combinations of 1, 3, and 17 starting values (i.e., installations) to initiate the experiments (Figure 2), which provided an analysis of how sensitive the results were to different amounts of prior information. We performed 5 replicates of each sensitivity analysis to determine how sensitive the results were to variations in each strategy. In sum, 30 sensitivity analyses were performed per strategy per experiment (180 total experiments).

### Multiarmed bandit strategies

The first strategy we used is deterministic probability matching (Figure 2A). This exploitative and so‐called “greedy” strategy always chooses to allocate the treatment with the highest probability of success when compared to other treatments per decision. Deterministic probability matching can be considered a passive adaptive‐management strategy because it allocates all available treatment deployments to what appears to be the current “best” option at each decision in sequence (Robbins 1956). In passive adaptive management, learning is used to make decisions but is considered an untargeted byproduct of maximizing the treatment performance (Williams et al. 2009). This and other greedy strategies are easy to implement, and they are adaptive in the sense that they exploit prior information to maximize performance (Scott 2010; Chapelle and Li 2011; Villar et al. 2015; Sutton and Barto 2018).

Randomized probability matching (Figure 2B) is an example of an active adaptive‐management strategy. This strategy allocates treatments in proportion to their probability of success per decision. Randomized probability matching is among the oldest multiarmed bandit strategies to inform active adaptive management (Thompson 1933). We used this strategy because it is simple to understand by practitioners with little experience with these concepts, it does not require the full‐time frame for experimentation to be known (e.g., discounting future payoffs) (Gittins 1979), it does not require certain assumptions (e.g., known prior distribution) (Sutton and Barto 2018), and it has been shown to outperform other Bayesian and nonBayesian strategies in theoretical and empirical analyses (Scott 2010; Chapelle and Li 2011; Agrawal and Goyal 2012; Russo and Van Roy 2014). Randomized probability matching is also easy to apply in general adaptive management settings and tends to balance exploitation and exploration naturally, although it is not known to optimize any specific utility function (Scott 2010).

We compared the probability‐matching strategies to a naive equal‐allocation strategy (Figure 2C). The traditional approach to equal allocation is to equally allocate all treatment options at each decision in sequence until the probability of some treatment being successful exceeds some subjective threshold, after which the superior treatment is allocated exclusively (Scott 2010). Therefore, this approach most closely resembles a classical, nonsequential experiment. We emphasize a nonadaptive approach to equal allocation, in which equal allocation is continued over the entire experiment regardless of the probability of the success of the treatments. This strategy is expected to be inefficient in terms of minimizing N effluent concentration. Rather, the strategy emphasizes learning as a sole objective.

### Computing probabilities

Using Bayesian statistics, probability distributions are approximated by the accumulation of many random samples that form the “shape” of a probability distribution. For some families of distributions, the posterior distributions of treatment‐specific N effluent concentrations can be computed using conjugate priors. We used Markov Chain Monte Carlo because it is more flexible and easily implemented (Gelman et al. 2013).

For each treatment *i* we used a natural log‐normal likelihood with vague priors to describe N performance as: $\theta_{i}\;\sim \;\mathrm{LogNormal}(\mu_{i},\tau_{i}$), where *µ* is the mean N effluent concentration and *τ* is precision. We assumed vague priors of $\mu_{i}\;\sim \;\mathrm{Uniform}(-10, 10)$ and $\tau_{i}\;\sim \;\mathrm{Uniform}(0, 5)$ for all *i* to have little or no influence on the posteriors (Hobbs and Hooten 2015). Prior to the initial allocation of treatments, we specified varying amounts of starting values and calculated the posterior distribution of N load for each treatment. Following each allocation, new data were sampled, and the posterior distributions were again updated. We used Just Another Gibbs Sampler version 4.3.0 (Denwood 2016; Plummer et al. 2019a, 2019b) interfaced in R version 3.6.1 (Hobbs and Hooten 2015; R Core Team 2016). We assembled 3 chains of 10 000 samples per treatment per decision. We discarded 50 000 “burn‐in” samples before recording samples in a chain. We assured convergence of posterior distributions via visual inspection of trace plots (Hobbs and Hooten 2015) and via the diagnostics of Heidelberger and Welch (1983) and Gelman and Rubin (1992).

To compute the probability of success of each treatment per decision, we used the 10 000 samples from the posterior distributions for each treatment and, for each sample, identified which treatment had the minimum N concentration (Scott 2010). We then tallied the proportion of samples in which each treatment had the minimum N effluent concentration over all samples. These proportions represent the probabilities that each treatment is superior to the others and was used in the deterministic and randomized probability‐matching algorithms. Regarding equal allocation, we equally sampled each treatment at each decision. Since we allocated 10 installations at each decision, we randomly chose 1 treatment to allocate 4 installations and the other treatments to allocate 3 installations (Figure 2C). The R code for the case study is provided in the Supplemental Data.

## RESULTS

### Success probabilities

**Figure 3:**
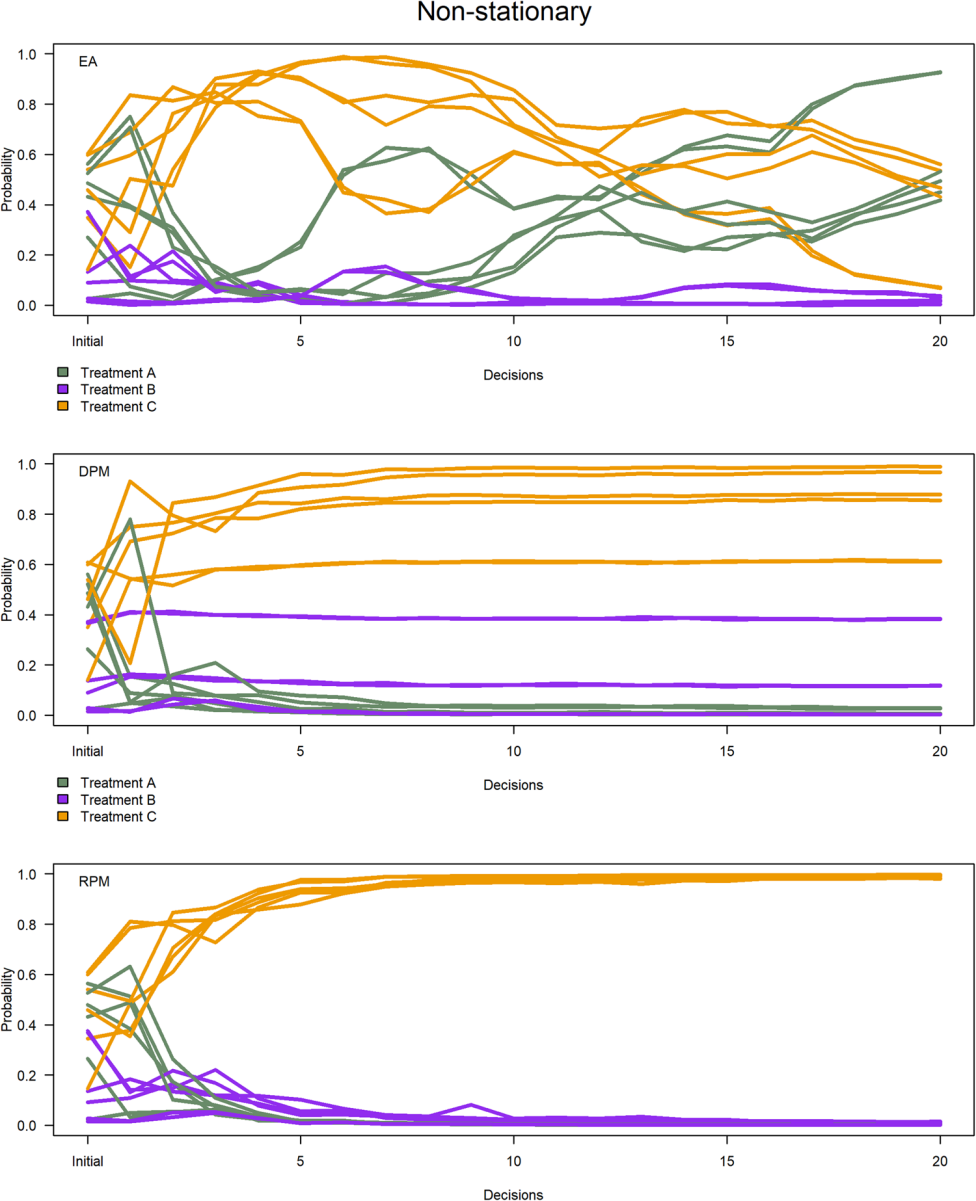
Probability of success per treatment per strategy. Each line represents mean probabilities across 5 replicate experiments. Six colored lines for each treatment represent sensitivity analyses with different starting values. The horizontal axis refers to initial probabilities to start experiments, followed by 20 decisions. *EA—equal allocation; DPM—deterministic probability matching; RPM—randomized probability matching.

**Figure 4:**
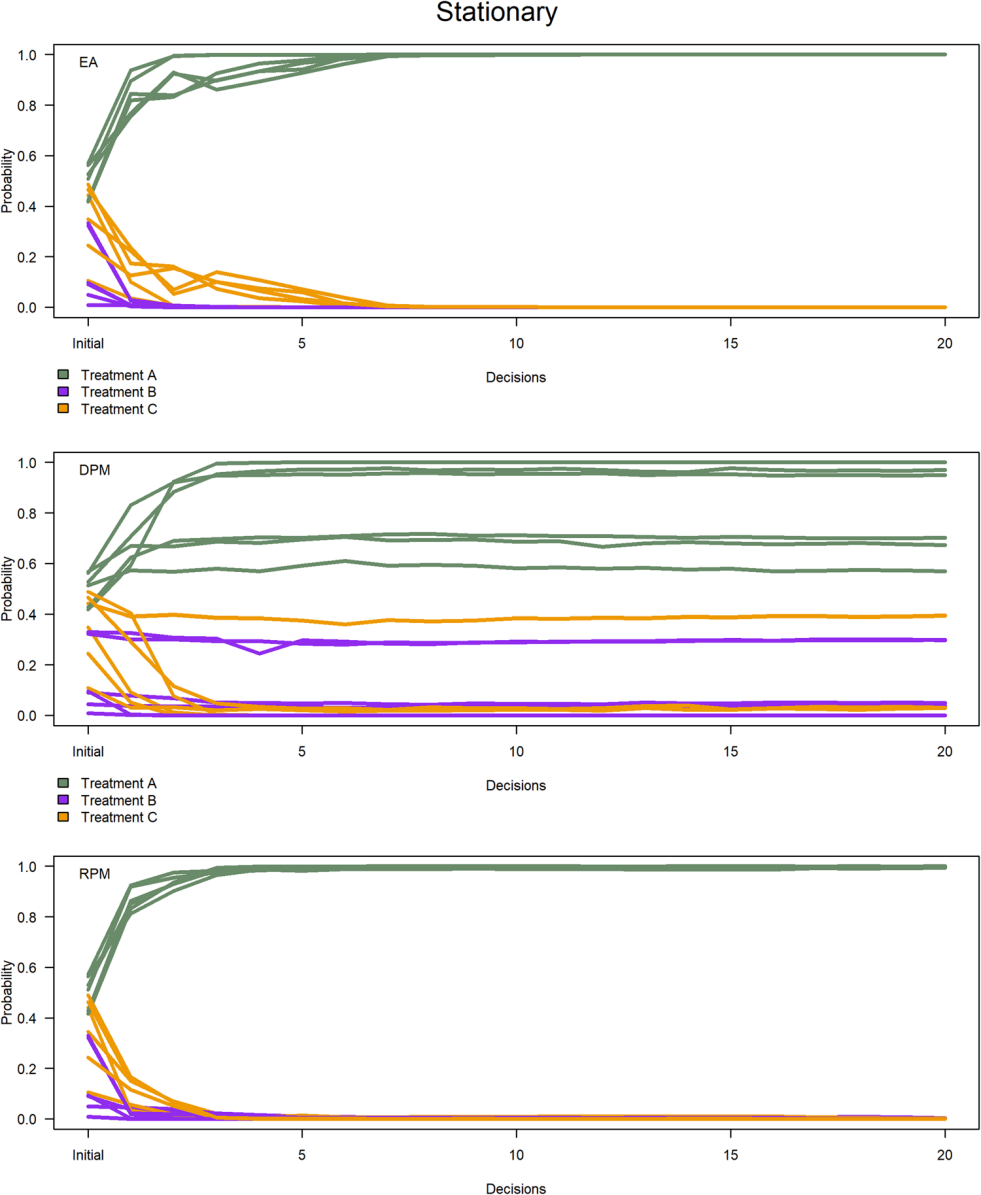
Probability of success per treatment per strategy. Each line represents mean probabilities across 5 replicate experiments. Six colored lines for each treatment represent sensitivity analyses with different starting values. The horizontal axis refers to initial probabilities to start experiments, followed by 20 decisions. *EA—equal allocation; DPM—deterministic probability matching; RPM—randomized probability matching.

The probability of success of each treatment varied in nonstationary situations (Figure 3), whereas Treatment A achieved a high probability of success in stationary situations (Figure 4). Recall from the treatment data that we assume Treatment A is the superior treatment regarding mean N effluent concentration over time. Success probabilities were more variable in the nonstationary context because the chronological sequence of Treatment A N effluent concentrations changed throughout the allocations. In the nonstationary context, Treatment A achieved high probabilities under 2 sensitivity analyses of equal allocation (Figure 3, upper panel) because the strategy sampled enough installations of Treatment A to recognize that mean N effluent concentration was changing. The other 4 sensitivity analyses showed an upward trend in Treatment A probabilities toward the end of the experiments (Figure 3, upper panel). The probability‐matching strategies did not sample enough installations of Treatment A to recognize its changing mean N effluent concentration in the nonstationary context (Figure 3, middle and lower panels).

Success probabilities were more consistent in the stationary context because Treatment A minimized N effluent concentration compared to the other treatments. Each sensitivity analysis of the randomized probability‐matching strategy achieved high probabilities faster than the other strategies (Figures 2 and 4, lower panel). All 6 sensitivity analyses of the strategy converged on Treatment A probabilities that were greater than 0.90 after 2 decisions (Figures 2B and 4, lower panel), whereas only 2 sensitivity analyses of deterministic probability matching achieved Treatment A probabilities that were greater than 0.90 altogether (Figure 4, middle panel). Inconsistent probabilities under deterministic probability matching were due to less‐informative starting values in the sensitivity analyses, as well as a lack of exploration of Treatments B and C (Figure 2A). Less‐informative starting values did not allow the posterior distributions of the inferior treatments to improve or tighten on the target distribution. Likewise, not enough installations of the inferior treatments were sampled to know that Treatment A was superior, which should have corresponded to a high probability of success.

All 6 sensitivity analyses of equal allocation achieved Treatment A probabilities of 1.0 after 8‐13 decisions in stationary situations (Figure 4, upper panel; see Supplementary Data). This result was due to excessive exploration of Treatments B and C that tightened the posterior distributions on the target distribution. In other words, learning was greater under the equal allocation strategy. It took longer to achieve high Treatment A probabilities (greater than 0.90) in 4 sensitivity analyses of equal allocation compared to randomized probability matching (Figure 4, upper panel) due to less‐informative starting values in the sensitivity analyses, as well as excessive exploration of inferior treatments (Figure 2C). Randomized probability matching controlled for these limitations in all sensitivity analyses (Figure 4, lower panel).

There was not a large variation in success probabilities across replicates of each sensitivity analysis (see Supplemental Data). Randomized probability matching and equal allocation had the largest variance in the first few decisions of each sensitivity analysis, as high as 0.05 and 0.001, respectively. Variance declined significantly to less than 0.000 01 for each strategy thereafter. Higher variance in early decisions of equal allocation and randomized probability matching was respectively due to the randomized allocation for each decision and the random features of the algorithm. The deterministic probability‐matching strategy maintained extremely low variance in probabilities over all replicates per experiment (less than 0.000 01).

### Cumulative performance

**Figure 5:**
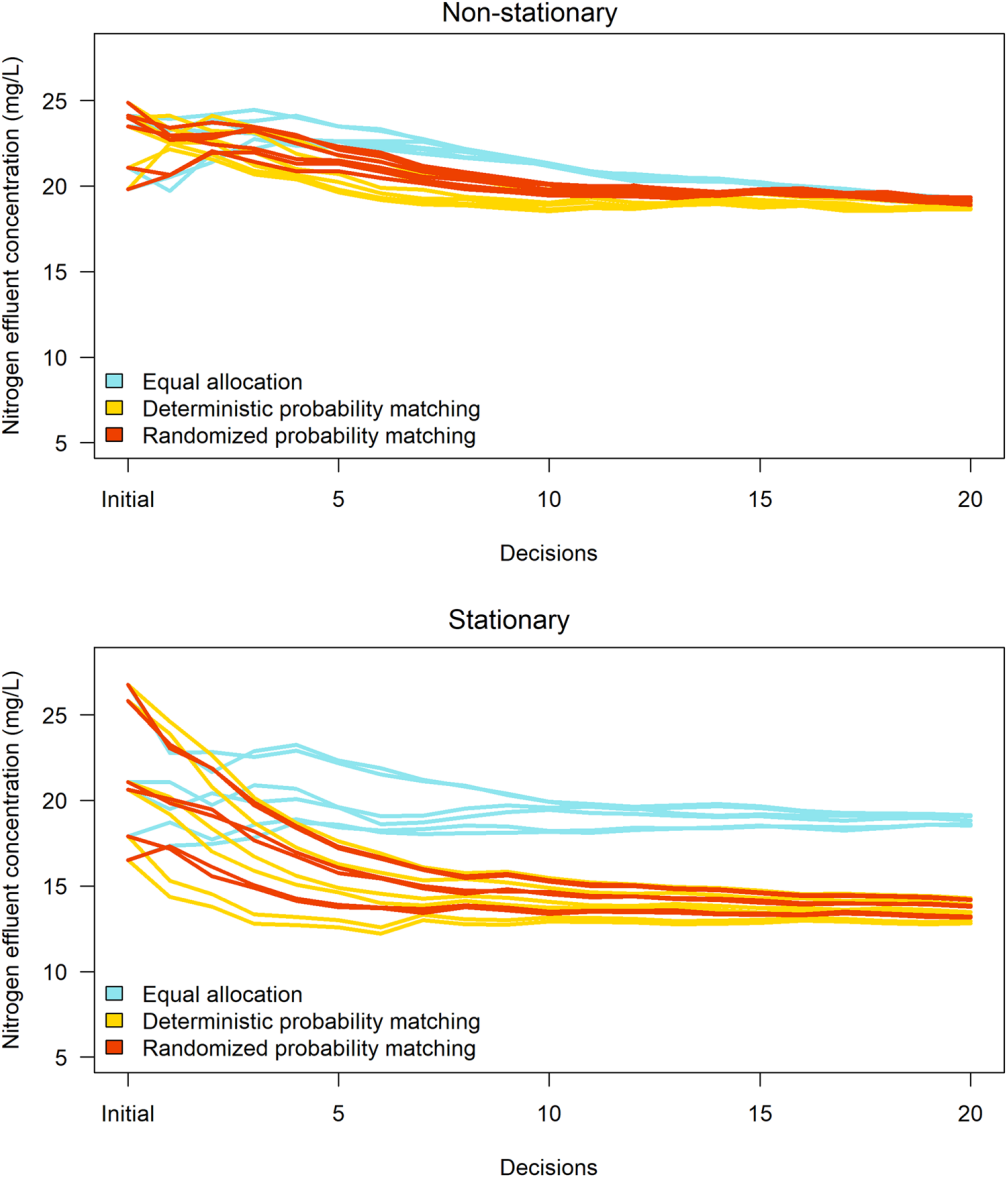
Cumulative performance per strategy. Each line represents mean N effluent concentration across 5 replicate experiments. Multiple lines for each strategy represent sensitivity analyses with different starting values. The horizontal axis refers to initial performance to start experiments, followed by 20 decisions.

The cumulative performance was recorded as the mean N effluent concentration (mg/L) over all treatment installations per decision per experiment. In the nonstationary context, the cumulative performance was worse in early decisions using equal allocation and randomized probability matching because the strategies explored all treatments (Figure 5, upper panel). Cumulative performance converged to between 18–20 mg/L toward the end of the nonstationary situations, with deterministic probability matching achieving slightly better performance over randomized probability matching and equal allocation (Figure 5, upper panel). Recall that the posterior distributions of the probability‐matching strategies converged on Treatment B (Figure 3, middle and lower panels), which is assumed to be an inferior treatment in the case study. The resulting treatment allocations were very similar among the strategies and, hence, cumulative performance did not vary by much. The mean of the total sum of installations throughout the experiments was 4212 mg/L under deterministic probability matching), 4231 mg/L under randomized probability matching, and 4250 mg/L under equal allocation. We presume that more decisions would allow the equal allocation strategy to converge on Treatment A, thereby minimizing N effluent concentration, but we could not test that inquiry due to data limitations. Likewise, we cannot concede the possibility that the probability‐matching strategies could converge on Treatment A given enough decisions or an infinite time horizon. In summary, there is not enough information to determine which strategy minimizes N effluent concentration in the nonstationary context.

In the stationary context, cumulative performance of equal allocation converged to approximately 19.5 mg/L, whereas the cumulative performance of the deterministic and randomized probability‐matching strategies declined significantly to approximately 13.5 mg/L and 13.7 mg/L, respectively (Figure 5, lower panel). The mean of the total sum of installations throughout the experiments was 2976 mg/L under deterministic probability matching, 3059 mg/L under randomized probability matching, and 4156 mg/L under equal allocation. Randomized probability matching slightly outperformed deterministic probability matching in 1 sensitivity analysis, by 0.3 mg/L, because less‐informative starting values led the deterministic probability‐matching strategy to choose Treatment C in the first decision of the experiment. Deterministic probability matching slightly outperformed randomized probability matching in the other 5 sensitivity analyses, by between 0.5–1.0 mg/L, because more‐informative starting values allowed deterministic probability matching to choose Treatment A to initiate the experiments. In summary, starting values in the sensitivity analyses are a determinant to variation in cumulative performance.

There was not a large variation in cumulative performance across replicates of each sensitivity analysis (see Supplemental Data). Randomized probability matching and equal allocation had the largest variance in the first few decisions of each sensitivity analysis, as high as 0.1 mg/L and 1.4 mg/L, respectively. After the first few decisions, variance in these strategies declined significantly to less than 0.05 mg/L for each strategy. Higher variance in early decisions of equal allocation and randomized probability matching was due to the randomized allocation of treatments and the random features of the algorithm, respectively. The deterministic probability‐matching strategy maintained no variance in treatment performance over all replicates because the same treatments were sampled per decision.

Results from each of the 180 experiments and summary statistics are provided in the Supplemental Data.

## DISCUSSION

The randomized probability‐matching strategy balanced exploitation with exploration in the stationary context. Deterministic probability matching exhibited more exploitative behavior in the stationary context (Figures 2A and 5, lower panel) because it bases allocations on what is known to be superior at each decision iteration. However, randomized probability matching balanced exploitation in performance and exploration in learning, which was comparable to deterministic probability matching and equal allocation. These findings have been defended in other experiments (Scott 2010; Chapelle and Li 2011; Russo and Van Roy 2014).

Differences in learning and performance favored randomized probability matching over equal allocation. This was primarily due to the exploitative features of randomized probability matching. Equal allocation tended to exhibit rapid learning that was comparable but slightly inferior to randomized probability matching in stationary situations (Figures 2B,C and 4, upper and lower panels). However, equal allocation exhibited poor performance (Figures 2C and 5, lower panel) due to excessive exploration of inferior treatments at each decision in the experiments. Likewise, equal allocation did not exploit Treatment A in any experiment due to its nonadaptive features.

Differences in learning and performance between the probability‐matching strategies are primarily due to the explorative features of randomized probability matching. Both probability‐matching strategies adequately exploited Treatment A in stationary situations (Figures 2A,B and 5, lower panel). However, deterministic probability matching exhibited variable success probabilities (Figure 4, middle panel) due to limited exploration of Treatments B and C. Exploration is most important in the first few decisions in an experiment. Randomized probability matching is an active strategy, meaning that it allocated inferior treatments in early decisions to improve the posterior distribution of Treatment A. In most of the sensitivity analyses, these allocations caused randomized probability matching to pace slightly behind deterministic probability matching regarding cumulative performance. This is attributable to the greater focus on exploration. With adequate exploration, however, randomized probability matching converged quickly on high success probabilities of Treatment A (Figures 2B and 4, lower panel) and therefore the cumulative performance of the strategy could trend toward mean Treatment A performance given an infinite time horizon.

The amount of N effluent reaching Cape Cod bays and estuaries in the years 2000 to 2018 could have been reduced by 28% under deterministic probability matching and 26% under randomized probability matching (Figure 5, lower panel). These findings could advance the implementation of probability‐matching strategies by conservation practitioners and state water quality managers to inform decisions on what treatments to install at each decision point through time. Exploration will be more important when uncertain innovations are tested. Practitioners can concurrently maintain some exploitation of treatments that have accumulated partial information on their performance. The algorithms contained in the probability‐matching strategies can aid in converging on the better treatment(s) in the long term and the results provide evidence of this. State managers can monitor long‐term estuary properties with full confidence that practitioners are allocating treatments in an efficient and statistically robust manner.

The primary limitation of this research concerns what to do with nonstationarity in sequential decision making. Equal allocation is an aggressive approach to learning and, as such, necessarily sacrifices short‐term performance. Its nonadaptive features could be useful for practitioners under nonstationary contexts because it does not require prior information on the treatments. Prior causal information could be useful in nonstationary contexts to determine what can be done to prevent degradation in treatment or for situations where confounding variables impact decision effectiveness. In the nonstationary context, the probability‐matching strategies converged on Treatment C, which is an inferior treatment in the case study. Deterministic probability matching did not perform enough exploration of Treatments A or B because the posterior distributions did not tighten on the target distributions. In general, passive adaptive management strategies like deterministic probability matching could converge on inferior treatments without knowing they are inferior (Walters and Holling 1990).

Randomized probability matching could converge on superior treatments and potentially outperform the other strategies in some nonstationary situations. The reasons for this are 2‐fold. First, the stochastic features of the strategy allow the opportunity to converge on superior treatments because the algorithm produces the occasional allocation to lower‐probability treatments. In the case study, the algorithm occasionally allocated to Treatment A based on extremely low probabilities, as low as 0.002 (see Supplemental Data). Although the learning ability at this stage of experimentation might be relatively low, there is still the ability to learn and potentially converge on a high‐performing treatment. We could not prove this potential outcome in the case study due to data limitations and the rate at which N effluent concentrations of Treatment A changed in the nonstationary context. However, theoretical experiments provided evidence that randomized probability matching can perform better than other multiarmed bandit strategies in nonstationary contexts (Gupta et al. 2011; Agrawal and Goyal 2012).

**Figure 6:**
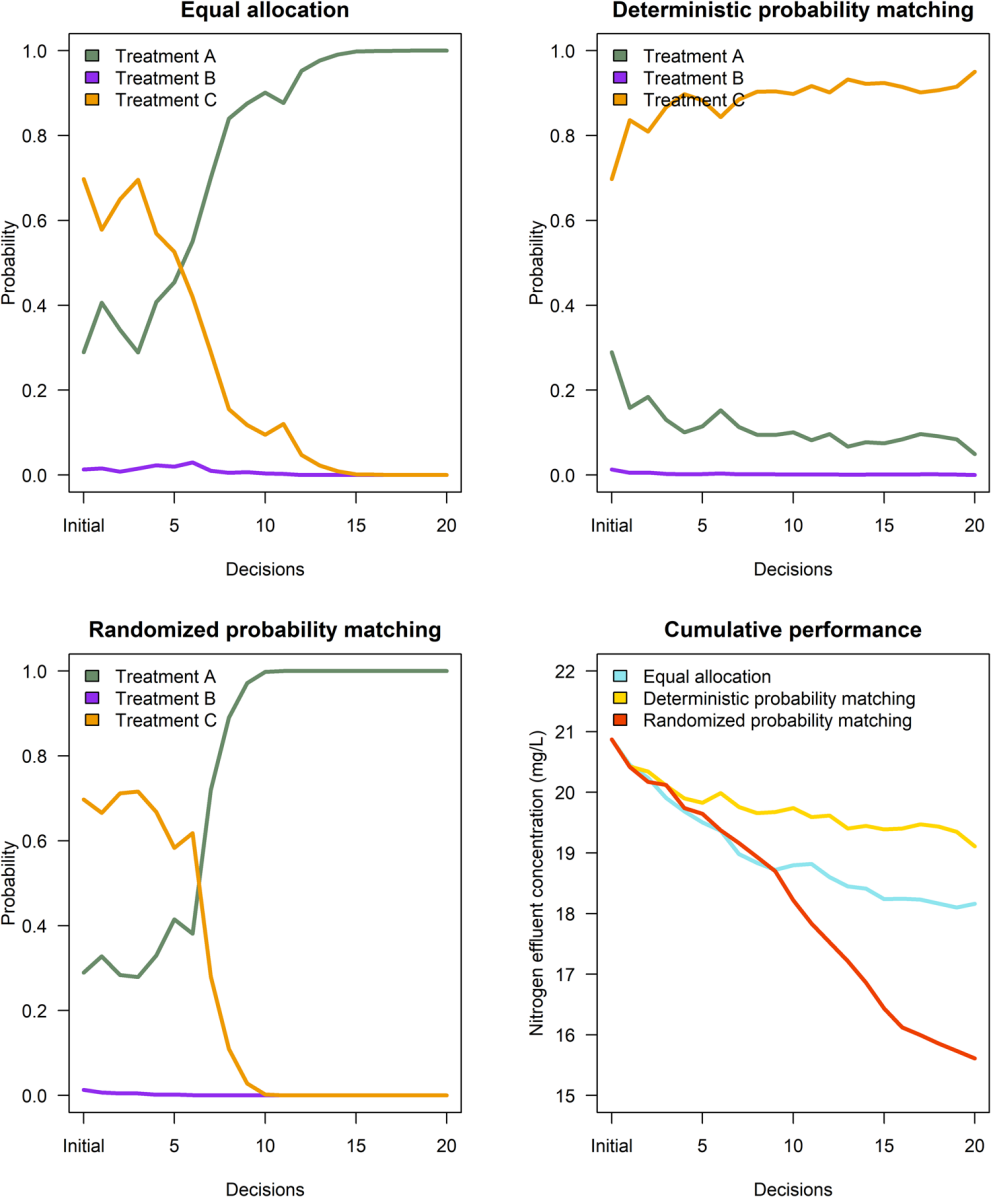
Nonstationary results from sensitivity analysis with 50 starting values per treatment. The horizontal axis refers to the initial probabilities to start experiments, followed by 20 decisions. Upper left, upper right, and lower left panels show the probability of success per strategy. The lower right panel shows mean N effluent concentration per strategy.

Second, mean N effluent concentration can change over time for several reasons, including chance, postinstallation improvements in technology, and uncontrolled environmental conditions. The case study data sets were developed to generalize to situations where stationarity might not be assumed. In these situations, different amounts of prior information and the rate at which treatments change over time dictate whether randomized probability matching can converge on high‐performing treatments. To illustrate this point, recall that the sensitivity analyses had different combinations of 1, 3, and 17 starting values to initiate the experiments. For the sake of example, we ran 1 nonstationary sensitivity analysis with 50 starting values for each treatment. In other words, we assumed a significant amount of partial information on each treatment, and a higher rate at which Treatment A, the potentially superior treatment, changes over the experiment. Results show that randomized probability matching achieved a higher probability of success and cumulative performance compared to equal allocation (Figure 6). There was a higher rate of learning using randomized probability matching (Figure 6, lower left panel) versus equal allocation (Figure 6, upper left panel). Likewise, randomized probability matching minimized N effluent concentration compared to the other strategies (Figure 6, lower right panel). Deterministic probability matching converged on Treatment C (Figure 6, upper right panel), which shows the potential irrelevancy of this strategy in nonstationary contexts. Based on this result, it is ambitious to presume that equal allocation is the better strategy in all nonstationary contexts, especially when the rate of treatment N effluent concentration changes over time.

These findings have important implications for the risk tolerance of practitioners and state water quality managers. Any approach to adaptive management may be less effective than otherwise if the central tendencies of probability distributions are changing faster than the rate of learning (Johnson et al. 2002). In stationary situations, the results provide evidence of a potential downside risk of underperformance when using a nonadaptive strategy due to excessive exploration and limited exploitation (Figures 2C and 5, lower panel). In nonstationary situations, the main downside risk of underperformance is allocating potentially inferior treatments that people don't know are inferior. If the probability distributions of treatments change rapidly, practitioners do not have enough time to monitor and identify the distributions before they have changed again. Nonstationarity is an often‐overlooked challenge in adaptive management. The challenge becomes increasingly problematic given the accelerating pace of global change and its effects on state‐dependent environmental outcomes (Williams and Brown 2014).

The critical relevance of this study is based on practitioners and state managers following a formal decision‐making process and having an a priori choice of strategy that can reduce uncertainty and maximize performance. Lacking sufficient prior information and long, nonstationary time series can pose challenges for adaptive management. However, the results of this study provide evidence that randomized probability matching can outperform the other strategies in both stationary and nonstationary contexts. The results did not show that the other strategies can outperform randomized probability matching in both stationary and nonstationary contexts. In consideration of these findings, we recommend the following flow of work to implement total maximum daily load policies: 1) calculate posterior distributions for each viable treatment as observational (monitoring) data is acquired; 2) use samples of the posterior distribution to assess the probabilities that each treatment is “best”; 3) decide to implement new treatments according to multiarmed bandit strategies; 4) acquire monitoring data; and 5) repeat.

## CONCLUSIONS

This article demonstrates that probability‐matching strategies are useful to inform adaptive management, especially water quality problems where the effects of actions are uncertain and decision making is not currently dependent on the state of ecosystems. Under a stationary context in which mean N effluent concentration does not change over time, both passive and active probability‐matching strategies outperformed the nonadaptive equal allocation strategy. Randomized probability matching tended to enhance learning without significant reductions in performance, as compared to deterministic probability matching. Although nonstationarity poses challenges to adaptive management, randomized probability matching can balance performance with learning in some nonstationary contexts.

It is important to note that the findings are meaningful to other similar problems. For example, the best herbicide for treating an invasive plant species or the best constant harvest rate for a huntable animal species may not depend on the abundance of the species. These situations may be due to variations in abundance that would not affect the actions chosen. Practitioners can utilize probability‐matching strategies that incorporate partial information on herbicide or harvest rate performance into informed choices in a similar way as we have shown in the case study.

The case study provided evidence of the usefulness of probability‐matching strategies to inform adaptive management on Cape Cod. Water quality in Cape Cod bays and estuaries could look different today if these strategies were implemented 20 years ago. Moving forward, practitioners can utilize these findings and related statistical methods to test and implement innovations to wastewater treatment and make better adaptive decisions over time. Some studies have suggested that an adaptive management strategy that has sufficient predictive capabilities can guard against many external effects and potential nonstationarity faced by environmental managers (e.g., Gregory et al. 2006). Our research on probability‐matching strategies could encourage additional research and wider application in traditional natural resource management.

## SUPPLEMENTAL DATA

ESM 1. R code for implementing experiments.

ESM 2. Experiment results.

## Supporting information

This article contains online‐only Supplemental Data.

Supporting information.

Supporting information.

## Data Availability

Data and associated metadata and calculation tools are available upon request by contacting corresponding author David M Martin (David.Martin@tnc.org).

## References

[ieam4302-bib-0001] Adler RW , Landman JC , Cameron DM . 1993 The clean water act 20 years later. Washington (DC): Island Press 333 p.

[ieam4302-bib-0002] Agrawal S , Goyal N . 2012. Analysis of Thompson sampling for the multi‐armed bandit problem. In: Proceedings of the 25th Annual Conference on Learning Theory. Edinburgh (UK): Association for Computational Learning. p 39.1–39.26.

[ieam4302-bib-0003] Amador JA , Görres JH , Loomis GW , Lancellotti BV . 2018 Nitrogen loading from onsite wastewater treatment systems in the Greater Narragansett Bay (Rhode Island, USA) watershed: Magnitude and reduction strategies. Water Air Soil Pollut 229(65).

[ieam4302-bib-0045] Barnstable County . 2020 Data and statistics. Barnstable County septic management program. Barnstable (MA): Barnstable County Department of Health and Environment. [accessed 2020 Jun 8]. https://septic.barnstablecountyhealth.org/category/data-and-statistics

[ieam4302-bib-0004] Boyd J . 2000 The new face of the clean water act: A critical review of EPA's proposed TMDL rules. Resources for the Future Working Paper No. 00‐12. [accessed 2019 Oct 28]. 10.2139/ssrn.215149

[ieam4302-bib-0005] Cai Q , Filos‐Ratsikas A , Tang P , Zhang Y . 2018. Reinforcement mechanism design for e‐commerce. In: Thirty‐Second AAAI Conference on Artificial Intelligence (AAAI‐18); 2018 Feb 2–7; New Orleans, LA. p 957–964.

[ieam4302-bib-0006] Cape Cod Commission . 2015 Cape Cod area wide water quality management plan update. Barnstable (MA): Cape Cod Commission. 254 p. [accessed 2019 Oct 29]. https://sp.barnstablecounty.org/ccc/public/Documents/208%20Final/Cape_Cod_Area_Wide_Water_Quality_Management_Plan_Update_June_15_2015-Printable.pdf

[ieam4302-bib-0007] Chapelle O , Li L . 2011 An empirical evaluation of Thompson sampling. Adv Neural Inf Process Syst 24:2249–2257.

[ieam4302-bib-0046] Commonwealth of Massachusetts . 2020 Approved Title 5 innovative/alternative technologies. [accessed 2020 Jun 8]. https://www.mass.gov/guides/approved-title-5-innovativealternative-technologies

[ieam4302-bib-0008] Denwood MJ . 2016 runjags: An R package providing interface utilities, model templates, parallel computing methods and additional distributions for MCMC models in JAGS. J Stat Softw 71(9):1–25.

[ieam4302-bib-0009] Eckles D , Kaptein M . 2019 Bootstrap Thompson sampling and sequential decision problems in behavioral sciences. Sage Open. 10.1177/2158244019851675

[ieam4302-bib-0011] Gelman A , Carlin JB , Stern HS , Dunson DB , Vehtari A , Rubin DB . 2013 Bayesian data analysis. 3rd ed. New York (NY): Chapman and Hall 675 p.

[ieam4302-bib-0010] Gelman A , Rubin DB . 1992 Inference from iterative simulation using multiple sequences. Stat Sci 7(4):457–511.

[ieam4302-bib-0012] Gittins JC . 1979 Bandit processes and dynamic allocation indices. J Roy Stat Soc B 41:148–177.

[ieam4302-bib-0013] Gregory R , Ohlson D , Arvai J . 2006 Deconstructing adaptive management: Criteria for applications to environmental management. Ecol Appl 16(6):2411–2425. 17205914 10.1890/1051-0761(2006)016[2411:damcfa]2.0.co;2

[ieam4302-bib-0014] Gunderson L , Light SS . 2006 Adaptive management and adaptive governance in the everglades ecosystem. Policy Sci 39:323–334.

[ieam4302-bib-0015] Gupta N , Granmo O , Agrawala A . 2011. Thompson sampling for dynamic multi‐armed bandits. In: Proceedings of the 10th International Conference on Machine Learning and Applications. Honolulu (HI): IEEE Computer Society. p 484–489.

[ieam4302-bib-0016] Heidelberger P , Welch P . 1983 Simulation run length control in the presence of an initial transient. Oper Res 31(6):1109–1044.

[ieam4302-bib-0017] Hobbs NT , Hooten MB . 2015 Bayesian models: A statistical primer for ecologists. Princeton (NJ): Princeton Univ Press. 320 p.

[ieam4302-bib-0019] Johnson FA , Kendall WL , Dubovsky JA . 2002 Conditions and limitations on learning in the adaptive management of mallard harvests. Wildlife Soc B 30(1):176–185.

[ieam4302-bib-0020] Kingsford RT , Biggs HC . 2012 Strategic adaptive management guidelines for effective conservation of freshwater ecosystems in and around protected areas of the world. Syndey (AU): IUCN WCPA Freshwater Taskforce, Australian Wetlands and Rivers Centre 43 p.

[ieam4302-bib-0021] Lai TL , Robbins H . 1985 Asymptotically efficient adaptive allocation rules. Adv Appl Math 6(1):4–22.

[ieam4302-bib-0022] Lancellotti BV , Loomis GW , Hoyt KP , Avizinis E , Amador JA . 2017 Evaluation of nitrogen concentration in final effluent of advanced nitrogen‐removal onsite wastewater treatment systems (OWTS). Water Air Soil Pollut 228(383).

[ieam4302-bib-0023] Martin DM , Johnson FA . 2019 Incorporating uncertainty and risk into decision making to reduce nitrogen inputs to impaired waters. J Environ Manag 249(109380). 10.1016/j.jenvman.2019.109380 PMC7901712 31434050

[ieam4302-bib-0024] Martin DM , Piscopo AN , Chintala MM , Gleason TR , Berry W . 2019 Structured decision making to meet a national water quality mandate. J Am Water Resour As 55(5):1116–1129. 10.1111/1752-1688.12754 PMC7859890 33551634

[ieam4302-bib-0025] McCarthy MA , Possingham HP . 2007 Active adaptive management for conservation. Conserv Biol 21(4):956–963. 17650246 10.1111/j.1523-1739.2007.00677.x

[ieam4302-bib-0026] National Research Council . 2001 Assessing the TMDL approach to water quality management. Washington (DC): National Academies Press. [accessed 2019 Oct 28]. 10.17226/10146

[ieam4302-bib-0027] Plummer M , Best N , Cowles K , Vines K . 2019a coda: Convergence diagnosis and output analysis for MCMC. R News 6(1):7–11.

[ieam4302-bib-0028] Plummer M , Stukalov A , Denwood M . 2019b rjags: Bayesian graphical models using MCMC. CRAN. p 4–9. [accessed 2020 Jun 8]. https://rdrr.io/cran/rjags/man/rjags-package.html

[ieam4302-bib-0029] R Core Team . 2016 A language and environment for statistical computing. Vienna (AT): R Foundation for Statistical Computing. [accessed 2020 Jun 8]. https://www.R-project.org/

[ieam4302-bib-0030] Rafferty AN , Ying H , Williams J . 2019 Statistical consequences of using multi‐armed bandits to conduct adaptive educational experiments. J Educ Data Mining 11:47–79.

[ieam4302-bib-0031] Robbins H . 1952 Some aspects of the sequential design of experiments. Bull Amer Math Soc 58(5):527–535.

[ieam4302-bib-0032] Robbins H . 1956 A sequential decision problem with a finite memory. Proc Natl Acad Sci USA 42(12):920–923. 16589976 10.1073/pnas.42.12.920 PMC528372

[ieam4302-bib-0033] Russo D , Van Roy B . 2014 Learning to optimize via posterior sampling. Math Oper Res 39:949–1348.

[ieam4302-bib-0034] Scott SL . 2010 A modern bayesian look at the multi‐armed bandit. Appl Stoch Model Bus 26:639–658.

[ieam4302-bib-0035] Springborn MR . 2014 Risk aversion and adaptive management: Insights from a multi‐armed bandit model of invasive species risk. J Environ Econ Manag 68(2):226–242.

[ieam4302-bib-0036] Sutton RS , Barto AG . 2018 Reinforcement learning: An introduction. 2nd ed. Cambridge (MA): MIT Press 552 p.

[ieam4302-bib-0037] Thompson WR . 1933 On the likelihood that one unknown probability exceeds another in view of the evidence of two samples. Biometrika 25(3/4):285–294.

[ieam4302-bib-0038] Villar SS , Bowden J , Wason J . 2015 Multi‐armed bandit models for the optimal design of clinical trials: Benefits and challenges. Stat Sci 30(2):199–215. 27158186 10.1214/14-STS504 PMC4856206

[ieam4302-bib-0039] Walters CJ . 1986 Adaptive management of renewable resources. New York (NY): Macmillan 374 p.

[ieam4302-bib-0040] Walters CJ , Holling CS . 1990 Large‐scale management experiments and learning by doing. Ecology 71(6):2060–2068.

[ieam4302-bib-0041] Williams BD , Szaro RC , Shapiro CD . 2009. Adaptive management: The US department of the interior technical guide. Washington (DC): Adaptive Management Working Group, US Department of the Interior. 72 p.

[ieam4302-bib-0042] Williams BK . 2011 Passive and active adaptive management: Approaches and an example. J Environ Manag 92(5):1371–1378. 10.1016/j.jenvman.2010.10.039 21074930

[ieam4302-bib-0043] Williams BK , Brown ED . 2012. Adaptive management: The US department of the interior applications guide. Washington (DC): Adaptive Management Working Group, US Department of the Interior. 136 p.

[ieam4302-bib-0044] Williams BK , Brown ED . 2014 Adaptive management: From more talk to real action. Environ Manag 53:465–479. 10.1007/s00267-013-0205-7 PMC4544568 24271618

