# Supplementary material for: A Multiarmed Bandit Approach to Adaptive Water Quality Management
Source: Integr Environ Assess Manag. 2020 Aug 14;16(6):841–52. doi: 10.1002/ieam.4302 (PMC7689691; doi:10.1002/ieam.4302)
Supplement: Supplementary file 1 — Supporting information. [file IEAM-16-841-s001.docx]

Supplemental information for “A multi-armed bandit approach to adaptive water quality management.”

| **Algorithm 1** R code for Markov Chain Monte Carlo sampling |
| --- |
| # call coda, rjags, runjags packages |
| library(coda) |
| library(rjags) |
| library(runjags) |
| # direction of preference for the objective |
| max.fcn = function(x) which(x==min(x)) |
| # empirical data |
| A = c() # Treatment A nitrogen concentrations (prior to each decision point) |
| nA = length(A) |
| B = c() # Treatment B nitrogen concentrations (prior to each decision point) |
| nB = length(B) |
| C = c() # Treatment C nitrogen concentrations (prior to each decision point) |
| nC=length(C) |
| # prepare data for Just Another Gibbs Sampler |
| jags.data = list(A=A,nA=nA,B=B,nB=nB,C=C,nC=nC) |
| # set initial values |
| init <- function(){list(muA=4,muB=4,muC=4)} |
| # parameters |
| parms = c('muA','tauA','muB','tauB','muC','tauC') |
| # model |
| run.mod <- "model{ |
| # prior probability distributions |
| muA ~ dunif(-10,10) |
| sigmaA ~ dunif(0,5) |
| tauA <- 1/sigmaA^2 |
| muB ~ dunif(-10,10) |
| sigmaB ~ dunif(0,5) |
| tauB <- 1/sigmaB^2 |
| muC ~ dunif(-10,10) |
| sigmaC ~ dunif(0,5) |
| tauC <- 1/sigmaC^2 |
| # likelihood distributions |
| for (i in 1:nA) { |
| A[i] ~ dlnorm(muA,tauA) |
| } |
| for (i in 1:nB) { |
| B[i] ~ dlnorm(muB,tauB) |
| } |
| for (i in 1:nC) { |
| C[i] ~ dlnorm(muC,tauC) |
| } |
| }" |
| n = 10000 # posterior sample size |
| c = 3 # number of chains |
| b = 50000 # discard burn-in sample |
| run.out <- run.jags(model = run.mod, monitor = parms, data = jags.data, inits = init, n.chains = c,burnin = b,sample = n,summarise=TRUE, method='rjags') |
| # summary results for each treatment |
| run.out |
| # assemble 30,000 posterior samples of each treatment into a matrix |
| ests = as.mcmc.list(run.out,vars=c('muA','muB','muC')) |
| mus1 = cbind(ests[[1]][,1],ests[[1]][,2],ests[[1]][,3]) |
| mus2 = cbind(ests[[2]][,1],ests[[2]][,2],ests[[2]][,3]) |
| mus3 = cbind(ests[[3]][,1],ests[[3]][,2],ests[[3]][,3]) |
| mus = rbind(mus1,mus2,mus3) |
| # diagnostics to assure convergence |
| plot(run.out) # trace plots and autocorrelation |
| gelman.diag(run.out) # Gelman and Rubin (1992) |
| heidel.diag(ests) # Heidelberger and Welch (1983) |

| **Algorithm 2** R code for posterior probabilities |
| --- |
| # determine frequency of times that each treatment minimizes performance out of 30,000 samples |
| x=apply(mus,1,max.fcn) |
| probability=numeric(3) |
| probability[1]=length(which(x==1))/(n*c) |
| probability[2]=length(which(x==2))/(n*c) |
| probability[3]=length(which(x==3))/(n*c) |
| probability |

| **Algorithm 3** R code for randomized probability matching |
| --- |
| ntrts = 10 # number of treatments to allocate |
| # draw random sample using probability |
| table(sample(c('A','B','C'),size=ntrts,replace=TRUE,prob=probability)) |
